# Optimal angle of needle insertion for spinal anesthesia in patients with spondylolisthesis: an ultrasonographic study

**DOI:** 10.1186/s12871-021-01444-0

**Published:** 2021-09-08

**Authors:** Youngwon Kim, Seokha Yoo, Sun-Kyung Park, Hansu Bae, Young-Jin Lim, Jin-Tae Kim

**Affiliations:** 1 Department of Anesthesiology and Pain Medicine, Seoul National University Hospital, Seoul National University College of Medicine, Daehak-ro, Jongno-gu, Seoul, Korea 03080; 2 Department of Anesthesiology and Pain Medicine, Dongguk University Ilsan Hospital, 27 Dongguk-ro, Ilsandong-gu, Goyang-si, Gyenggi-do Korea 10326

**Keywords:** Spinal anesthesia, Spondylolisthesis, Ultrasonography

## Abstract

**Background:**

Spondylolisthesis is a common degenerative spinal deformity. At the level of spondylolisthesis, the anatomy of the interlaminar space may differ from normal spine, in which case optimal angle of the needle insertion for spinal anesthesia may change. This study compared the optimal angle of needle insertion during spinal anesthesia in patients with and without lumbar spondylolisthesis using ultrasound.

**Methods:**

We recruited 40 patients, 20 with and 20 without lumbar spondylolisthesis (group S and N, respectively). Ultrasonography was performed in the transverse midline and parasagittal oblique views at the spondylolisthesis level and the adjacent upper level. We measured the probe application angle with the longest interlaminar height of the ligamentum flavum-dura mater complex (LFD), depth from the skin to the LFD, depth from the skin to the anterior complex, and intrathecal space width. A positive angle represented a cephalad angulation.

**Results:**

The optimal needle insertion angle in the transverse midline view at the spondylolisthesis level was (-) 2.7 ± 3.4° in group S and 0.8 ± 2.5° in group N (*P* $<$ 0.001). In the parasagittal oblique view, it was (-) 2.7 ± 4.5° in group S and 1.0 ± 3.2° in group N (*P* = 0.004). There were no between-group differences in the angles at the upper level, with all cephalad angles in both views. Other ultrasound image data were comparable between groups.

**Conclusion:**

In patients with spondylolisthesis, caudad angulation of the spinal needle can aid successful spinal puncture at spondylolisthesis level, both in the midline and paramedian approaches.

**Trial registration:**

www.ClinicalTrials.gov (NCT04426916); registered 11 June 2020.

## Background

Spinal anesthesia has been traditionally performed based on surface anatomical landmarks. Recently, however, with the widespread use of ultrasonography during spinal anesthesia, an approach based on individual anatomical characteristics has emerged [1, 2]. Ultrasonography facilitates spinal anesthesia, especially in patients with anatomical abnormality of spine, by providing characteristic anatomic information [3, 4].

Considering the angles of the spinous process and interlaminar space during lumbar flexion, it is recommended that the spinal needle should be inserted at a slight cephalad angle for successful spinal puncture [5]. However, it may not be possible to apply this approach in patients with spinal abnormalities such as spondylolisthesis. In patients with spondylolisthesis, the anatomy of the interlaminar space through which the needle passes during spinal anesthesia can be altered due to the angles formed by the two vertebral bodies and spinous processes [6]. Therefore, the optimal angle of needle trajectory in these patients may be different from that in the general population [7, 8].

Spondylolisthesis is commonly observed in the elderly undergoing spinal anesthesia [9]. However, the anatomical characteristics to be considered during spinal anesthesia in patients with spondylolisthesis have rarely been studied [10]. Ultrasound images can provide anatomical information that can guide the selection of the optimal angle and point of spinal needle insertion. The aim of this study was to determine whether there is a difference in the optimal angle of spinal needle insertion during spinal anesthesia between patients with spondylolisthesis and those without spondylolisthesis.

## Methods

### Study design

This trial was approved by the institutional review board of Seoul National University Hospital (No. 2005–149-1125) and written informed consent was obtained from all subjects participating in the trial. The trial was registered prior to patient enrollment at ClinicalTrials.gov (NCT04426916, Principal investigator: Jin-Tae Kim, Date of registration: 11 June 2020.)

From June to August 2020, we enrolled adult patients with an American Society of Anesthesiologists physical status of I–III who were scheduled for elective surgery at Seoul National University Hospital, Seoul, Republic of Korea. The first patient was enrolled in June, 23, 2020. Total of 40 participants, 20 patients with single-level spondylolisthesis (group S) and 20 patients with normal spinal anatomy (group N) were recruited. The sample size was set empirically by referring to previous studies comparing the distance on ultrasound images [11–13]. Lumbosacral spinal X-ray image were taken in all patients scheduled for surgery under spinal anesthesia for preoperative evaluation. Based on the preoperative X-ray images, patients with spondylolisthesis at only one lumbar level were screened and enrolled in group S. Patients without spondylolisthesis were enrolled in group N, and patients in group N were selected in a way that they were of the same gender, within 10% of the age, 10% of the height, and 15% of the weight of patients with spondylolisthesis. We excluded patients who had difficulty achieving the lateral decubitus position for spinal anesthesia, a history of lumbar spine surgery, serious spinal anatomical deformities other than spondylolisthesis, or spondylolisthesis at more than one level. All patients provided written informed consent prior to participation.

### Ultrasonography procedure

All participants underwent preoperative spinal ultrasonography in a curtained waiting room or an operating room. Ultrasonography was performed by an anesthesiologist (YK) with experience of performing ultrasound-assisted spinal anesthesia in more than 50 cases. The C5-2 s convex array (frequency range: 2–5 MHz) of a TE7 Touch Enabled Ultrasound System (Mindray, Shenzhen, China) or the C1-5 convex array of a Venue Go™ (GE Healthcare, Chicago, IL) was used for scanning. A pillow was placed under the patient’s head in the lateral decubitus position to align their spine and their knees were bent toward their chest with neck flexion to attain the best position for spinal anesthesia. Ultrasonography was performed in the midline transverse and paramedian sagittal oblique views at the level of the spondylolisthesis (e.g., L4 on L5, L4/5) and the adjacent upper level (e.g., L3/4); thus, four images were obtained for each patient.

**Fig. 1:**
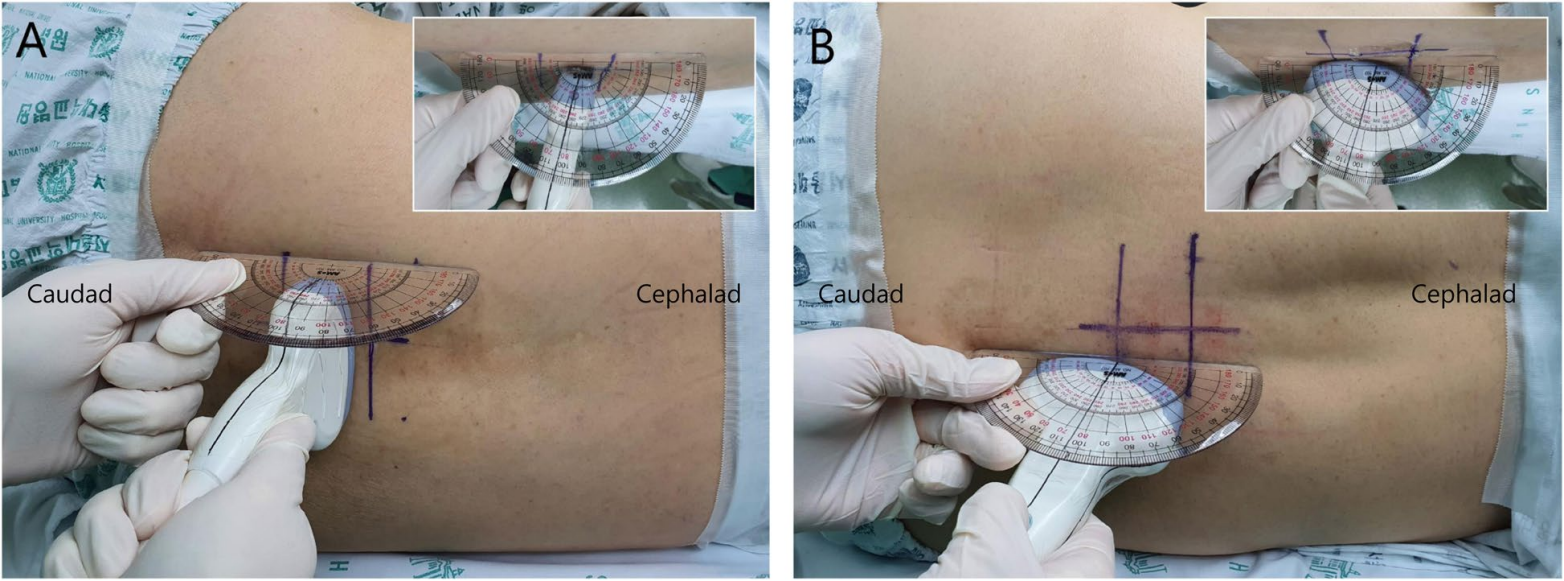
The method of angle measurement at transverse midline view and parasagittal oblique view. (**A**) The ultrasound probe was placed transverse to the patients' back and was tilted toward cephalad or caudad to find the longest observed the ligamentum flavum-dura mater complex (LFD) height. The probe application angle with the longest interlaminar height of LFD was measured using a protractor. The measured angle was ( +) 14° in this image. (**B**) The ultrasound probe was placed in the parasagittal plane with a medial tilt towards the midline. The probe is tilted toward cephalad to caudad, and the probe application angle with the longest interlaminar height of LFD was measured using a protractor. The measured angle was ( +) 15° in this image

**Fig. 2:**
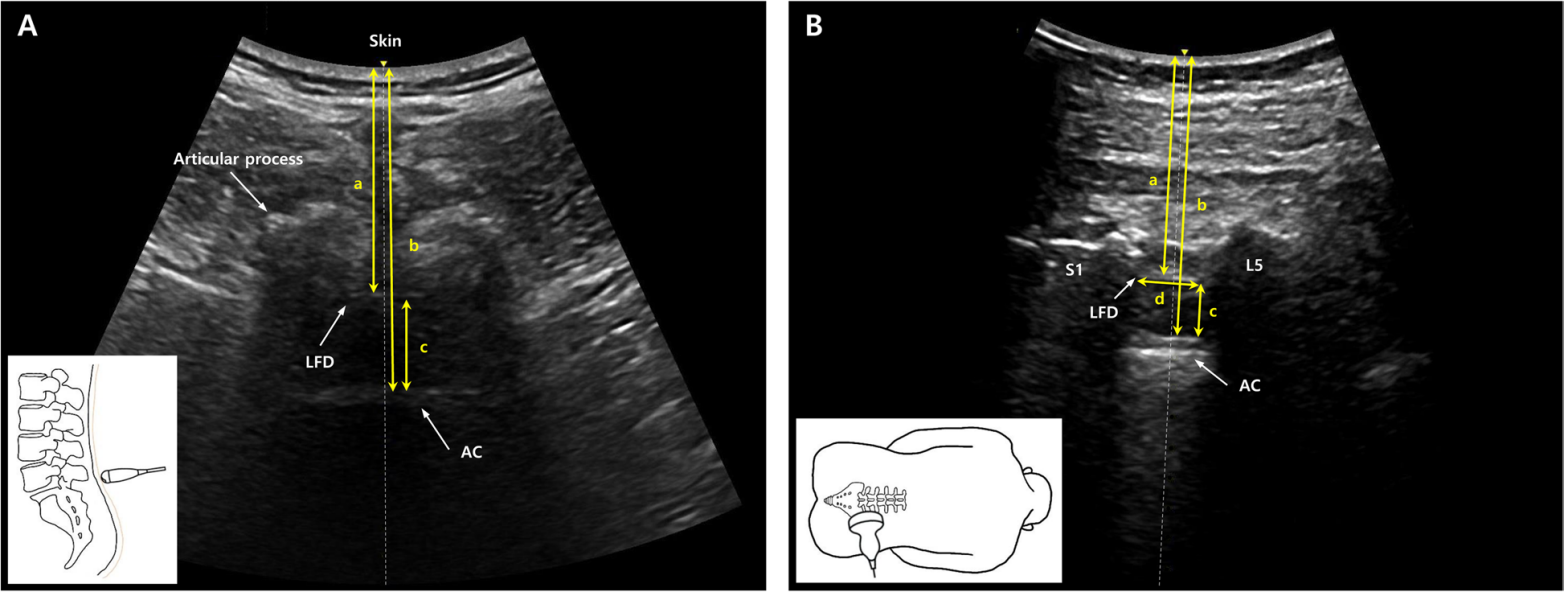
Spinal ultrasound image. (A) Spinal ultrasound image of transverse midline view of a spondylolisthesis patient at the level of L5-S1. As shown in the small schematic diagram at the left bottom of the ultrasound image, the image was taken with the ultrasound probe tilted toward caudad direction. (B) Spinal ultrasound image of parasagittal oblique view of a spondylolisthesis patient at the level of L5-S1. As shown in the small schematic diagram at the left bottom of the ultrasound image, the image was taken with the ultrasound probe tilted toward caudad direction, with a medial tilt to midline. LFD: Ligamentum flavum-dura mater complex. AC: Anterior complex. a: Depth from skin to the LFD. b: Depth from skin to the AC. c: Width of the intrathecal space (distance between LFD and AC). d: Interlaminar height of the LFD

First, we used the transverse midline view to identify and mark the tips of the spinous processes on the skin and drew the neuraxial midline by connecting the tips and determined the interspinous spaces. The probe was then placed at the middle of the interspinous space, where the anterior complex (AC) and LFD were visible in a plane perpendicular to the back. At the point, the probe was tilted cephalad or caudad with respect to perpendicular plane to determine the angle at which the LFD was the longest. This point and angle were regarded as the “expected optimal needle insertion point and angle” of transverse midline approach for successful spinal anesthesia. The angle between the central axis of the lateral face of ultrasound probe and the patient's back at the expected needle insertion point was measured using a protractor (Fig. 1). If the ultrasound probe was tilted cephalad at the longest observed LFD, the angle was marked as positive, and if it was tilted caudad, the angle was marked as negative. The ultrasonography image in the paramedian sagittal oblique view was obtained on the dependent side by placing the ultrasound probe lateral to the midline and tilting it medially. The level of each interlaminar space was confirmed by counting up from the sacrum [11]. The expected optimal needle insertion angle was measured using the same method that was used for the transverse midline view (Fig. 1). The probe was placed at where the AC and LFD were visible and was tilted cephalad or caudad to determine the angle at which the interlaminar height of the LFD, defined as the length of the hyperechoic line visible through the interlaminar space, was the longest. The angle between the central axis of the frontal face of probe and patient’s back was measured. In the patients in group N, the angle was measured at a level corresponding to the spondylolisthesis level of the patient in group S with whom they were matched. Using the obtained images, the interlaminar height of the LFD, depth from the skin to the LFD, depth from the skin to the AC, and width of the intrathecal space (distance between LFD and AC) were measured. Another anesthesiologist who was blinded to group allocation performed these measurements. Representative ultrasound images of the transverse midline and parasagittal oblique view with measurements are shown in Fig. 2.

### Outcome assessment

The primary outcome was the angle between the central axis of the ultrasound probe and the patient's back at the point at which the interlaminar space was widest in the midline transverse and paramedian sagittal oblique views at the level of the spondylolisthesis. Secondary outcomes included the angles measured using same method at the adjacent upper level of the spondylolisthesis and the interlaminar height of the LFD, depth from the skin to the LFD, depth from the skin to the AC, and width of the intrathecal space (distance between LFD and AC) at each level and view.

All methods were carried out in accordance with relevant guidelines and regulations.

### Statistical analysis

Continuous data were tested for normality using the Kolmogorov–Smirnov and Shapiro–Wilk tests. Normally distributed data were compared using the Student t-test (mean $\pm$ SD), and non-normally distributed data were compared using the Mann–Whitney test (median [interquartile range]). A two-tailed *P* < 0.05 was considered statistically significant. Categorical data were collated as numbers and percentages and compared using χ^2^ test or Fisher’s exact test. Data were analyzed using SPSS Statistics version 25.0 (IBM Corp., NY, USA).

## Results

A total of 40 patients (20 per group) were recruited for this study. The patients’ demographic characteristics are shown in Table 1. There were no differences between the two groups. All the patients in group S had grade 1 spondylolisthesis according to the Meyerding classification [14]. The spondylolisthesis levels were as follows: L3/4 in four, L4/5 in seven, and L5/S1 in nine patients.

**Table 1:** Patient demographics

	**No Spondylolisthesis (N = 20)**	**Spondylolisthesis (N = 20)**
Female sex, N	17 (85)	17 (85)
Age, yr	66.1 (9.7)	66.8 (9.9)
Height, cm	158.4 (8.0)	156.3 (7.5)
Weight, kg	64.0 (11.0)	64.6 (8.0)
BMI, kg/m^2^	25.4 (3.3)	26.4 (2.8)
ASA class, N		
I	5 [25]	6 (30)
II	13 (75)	12 (60)
III	2 [10]	2 [10]
**Comorbidities**, N		
Hypertension	11 (55)	11 (55)
Diabetes mellitus	3 (15)	4 (20)
Coronary artery disease	2 (10)	5 (25)
Cerebrovascular disease	1 (5)	1 (5)
Chronic liver disease	2(10)	2 (10)
Chronic kidney disease	3 (15)	1 (5)
Pulmonary disease	2 (10)	1 (5)
Others	3 (15)	0 (0)
**Spinal abnormality,** N		
Spondylosis	15 (75)	14 (70)
Scoliosis	5 (25)	5 (25)
Compression fracture	1 (5)	4 (20)
Other abnormality	7 (35)	7 (35)
**Level of spondylolisthesis, N**		
L3 on L4	-	4
L4 on L5	-	7
L5 on S1	-	9

Categorical data were compared using χ^2^ test, and presented as numbers and percentages

Continuous data presented as median (IQR) or number (%) of patients

The optimal angles of needle insertion at the level of the spondylolisthesis and the adjacent upper level are shown in Table 2. The mean of the optimal needle insertion angle at the level of the spondylolisthesis in the transverse midline view was (-) 2.7 ± 3.4° in group S and 0.8 ± 2.5° in group N (mean difference -3.6 (95% CI (-) 5.5 to (-) 1.6), *P* $<$ 0.001). In the parasagittal oblique view, the angle was (-) 2.7 ± 4.5° in group S and 1.0 ± 3.2° in group N (mean difference -3.7 (95% CI (-) 6.2 to (-) 1.2), *P* = 0.004). However, in both views, there were no between-group differences in the optimal angles of needle trajectory at the adjacent upper level of the spondylolisthesis. The angle was 1.3 ± 2.6° in group S and 1.8 ± 2.1° in group N (mean difference -0.5 (95% CI (-) 2.1 to 1.0), *P* = 0.49) in the transverse midline view, and it was 1.4 ± 3.9° in group S and 1.9 ± 1.6° in group N (mean difference 1.0 (95% CI (-) 2.4 to 1.5), *P* = 0.62) in the parasagittal oblique view.

**Table 2:** Optimal angle of needle trajectory at spondylolisthesis level and one upper level

	**Level**	**N**	**Normal**	**Spondylolisthesis**	*P* value
**Spondylolisthesis level**					
Angle in transverse midline view (**º)**	Total	20	0.8 ± 2.5	-2.7 ± 3.4	$<$ 0.001
	L3/4	4	1.5 ± 1.0	-2.5 ± 3.7	
	L4/5	7	2.6 ± 1.3	-1.7 ± 3.4	
	L5/S1	9	-0.9 ± 2.7	-3.1 ± 3.5	
Angle in Parasagittal oblique view (**º)**	Total	20	1.0 ± 3.2	-2.7 ± 4.5	0.004
	L3/4	4	2.0 ± 1.7	-1.0 ± 6.4	
	L4/5	7	2.7 ± 1.9	-1.7 ± 4.1	
	L5/S1	9	-0.8 ± 3.8	-4.4 ± 3.8	
**Upper level**					
Angle in transverse midline view (**º)**	Total	20	1.8 ± 2.1	1.3 ± 2.6	0.49
	L3/4	4	1.6 ± 2.0	1.9 ± 2.0	
	L4/5	7	2.6 ± 2.5	0.9 ± 2.8	
	L5/S1	9	1.3 ± 2.0	1.2 ± 3.0	
Angle in Parasagittal oblique view (**º)**	Total	20	1.9 ± 1.6	1.4 ± 3.9	0.62
	L3/4	4	2.0 ± 1.4	4.3 ± 2.2	
	L4/5	7	2.6 ± 1.7	0.9 ± 3.9	
	L5/S1	9	1.4 ± 1.7	0.6 ± 4.3	

Data presented as mean ± SD

P-values are the results of student-T test for continuous variables

**Fig. 3:**
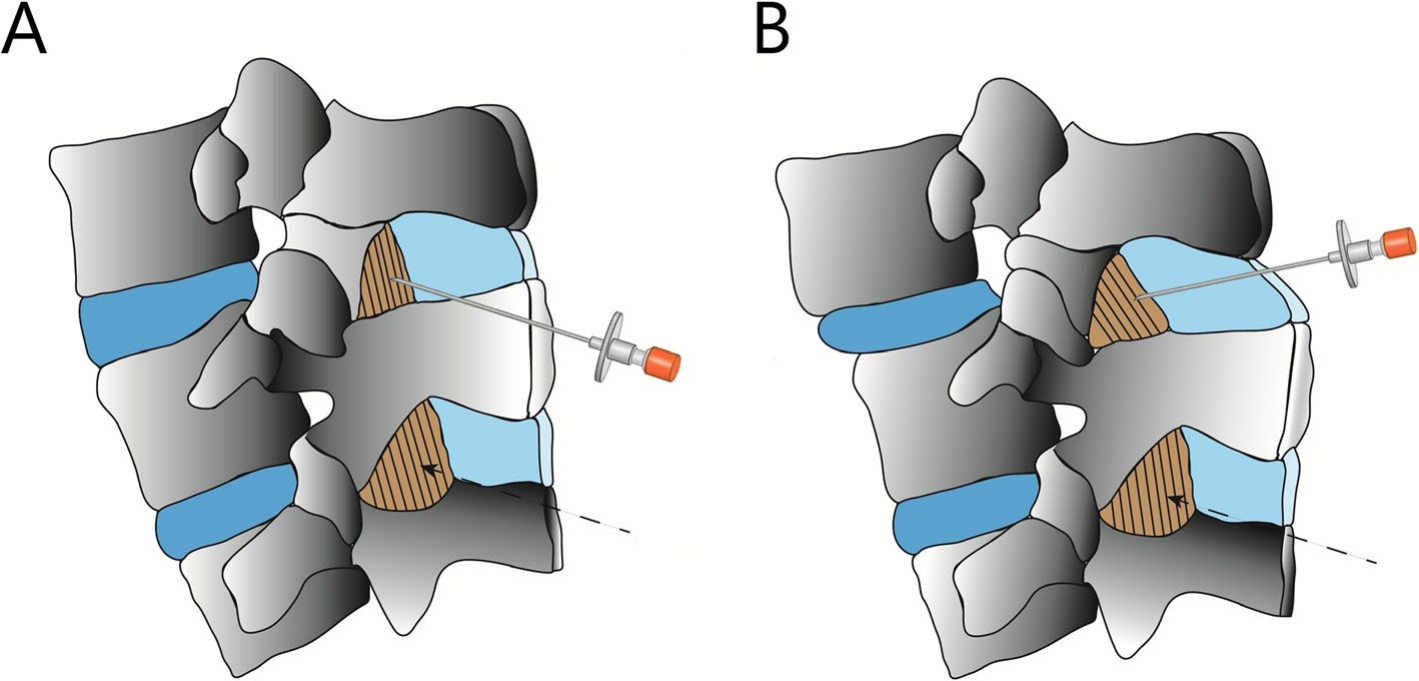
Schematic image of spinal anesthesia. Schematic image of trajectory of spinal needle to normal spine (**A**) and spine with lumbar spondylolisthesis (**B**). The figure represents parasagittal oblique approach of spinal anesthesia. Spinal needle is inserted in a cephalad direction for normal spine (**A**), while in a slightly caudal direction for spondylolisthesis level (**B**)

The interlaminar height of the LFD, depths from the skin to the LFD and to AC, and width of the intrathecal space were comparable between the two groups at both levels (Table 3). Figure 3 shows the schematic images representing the optimal angle of needle trajectory of groups N and S.

**Table 3:** The ultrasound data at spondylolisthesis level and one upper level

		**Normal**	**Spondylolisthesis**	*P* value
**Spondylolisthesis level**				
Transverse midline view	Depth to LFD, cm	4.2 ± 0.7	4.0 ± 0.6	0.50
	Depth to AC, cm	5.4 ± 0.6	5.3 ± 0.7	0.82
	LFD to AC, cm	1.2 ± 0.3	1.3 ± 0.3	0.22
Parasagittal oblique view	Interlamina height of LFD, cm	2.1 ± 0.9	1.8 ± 1.1	0.63
	Depth to LFD, cm	3.9 ± 0.5	3.8 ± 0.4	0.28
	Depth to AC, cm	5.1 ± 0.6	5.1 ± 0.6	0.67
	LFD to AC, cm	1.2 ± 0.2	1.3 ± 0.4	0.13
**Upper level**				
Transverse midline view	Depth to LFD, cm	4.1 ± 0.7	4.1 ± 0.6	0.83
	Depth to AC, cm	5.3 ± 0.7	5.5 ± 0.9	0.43
	LFD to AC, cm	1.2 ± 0.2	1.4 ± 0.5	0.16
Parasagittal oblique view	Interlamina height of LFD, cm	1.1 ± 0.4	1.0 ± 0.4	0.18
	Depth to LFD, cm	4.2 ± 0.5	4.3 ± 0.6	0.69
	Depth to AC, cm	5.6 ± 0.6	5.6 ± 0.8	0.68
	LFD to AC, cm	1.3 ± 0.4	1.3 ± 0.4	0.82

Data presented as mean ± SD

P-values are the results of student-T test for continuous variables

The depth from the skin to the LFD and AC and width of the intrathecal space at each level in group S are shown in Table 4. There were no statistically significant differences in the depths from skin to intrathecal space at the two levels.

**Table 4:** Depth from skin to subarachnoid space in spondylolisthesis patients

		Upper level	Spondylolisthesis level	P-value
Transverse midline	Depth to LFD (cm)	4.3 ± 0.6	4.0 ± 0.6	0.12
	Depth to AC (cm)	5.6 ± 0.8	5.3 ± 0.7	0.20
	LFD to AC (cm)	1.3 ± 0.4	1.3 ± 0.3	0.97
Parasagittal oblique	Depth to LFD (cm)	4.1 ± 0.6	3.8 ± 0.4	0.07
	Depth to AC (cm)	5.5 ± 0.9	5.1 ± 0.6	0.12
	LFD to AC (cm)	1.4 ± 0.4	1.3 ± 0.4	0.67

Data presented as mean ± SD

P-values are the results of student-T test for continuous variables

## Discussion

In this study, we used ultrasonography to evaluate the optimal angle of needle trajectory during spinal anesthesia in patients with spondylolisthesis. We found that the optimal spinal needle insertion at the level of the spondylolisthesis was in a caudad direction, contrary to that observed in patients without spondylolisthesis.

There have been many studies on anatomical considerations that facilitate spinal anesthesia in patients with lumbar spinal deformities; these studies have used imaging modalities such as computed tomography (CT), magnetic resonance imaging (MRI), and ultrasonography [11, 15]. Among these modalities, ultrasonography has proved its usefulness in visualizing the interlaminar space especially in patients with abnormal spinal anatomy [4, 16–18]. Spondylolisthesis is one of the most common degenerative spinal deformities, with an incidence of up to 30% in individuals over the age of 65 [19, 20]. To the best of our knowledge, this is the first ultrasonographic study on the optimal angle of needle insertion for spinal anesthesia in patients with spondylolisthesis. If possible, it would be better not to perform a spinal puncture at a level with spondylolysis. However, in older adults, spondylolisthesis is often accompanied at more than one lumbar level, and other spinal deformities can be accompanied at other levels. In such cases, the anesthesiologist might have to perform spinal puncture at a level with spondylolisthesis.

Conventionally, when performing spinal anesthesia using the midline or paramedian approach, the angle of needle insertion is often in a slightly cephalad direction according to the anatomy of the lumbar spinous process, which projects from the cephalad to the caudad direction. A previous study recommended a midline approach with a cranial angle of the needle to the dorsal plane of the skin of 90–110° [21]. and a cranial angle of the paramedian needle to the dorsal plane of the skin of 120–135° [22]. Vogt et al. analyzed the spine CT images of 52 patients without any structural anomaly and estimated that the optimal angles for needle insertion for spinal puncture at both the L3/4 and L4/5 levels were 9 degrees in the cephalad direction [23]. Another study by Puigdellívol-Sánchez [24] revealed that the angles from the axial plane to the skin passing above the inferior spinous process on the spinal MRIs of seven individuals were 9.0° and 8.9° at L3/4 and L4/5, respectively. However, our results showed that cephalad angulation of the spinal needle cannot be applied for spinal puncture at the level of the spondylolisthesis.

Since the upper vertebral body slides down along the superior endplate of the lower vertebral body in patients with spondylolisthesis, the angle of spinal needle insertion may need to be altered for successful spinal puncture (Fig. 3). According to our results, a caudad angulation is needed during needle insertion for spinal anesthesia at the level of the spondylolisthesis.

Notably, in both groups, the optimal needle insertion angle at the L5/S1 level tended to be more caudad-oriented than that at other levels, in both groups. Funao et al. [25] assessed the spinopelvic alignment of patients with and without spondylolisthesis using standing lateral radiographs. They reported that the sacral slope – the angle between the superior endplate of S1 and the horizontal plane – was much steeper than the L4 or L5 slope – the angle between the superior endplate of L4 or L5 and the horizontal plane – in groups with and without spondylolisthesis. Besides, Chen et al. retrospectively compared lumbar spine CT images between control and degenerative spondylolisthesis group, and found out that a large angle of sacral slope is a risk factor of L5 degenerative spondylosis [26]. As shown in previous researches, the steep angle of sacral slope may explain the more caudal angle of needle insertion at L5/S1 level.

There were no between-group differences in the interlaminar height of the LFD and width of the intrathecal space, regardless of the level. This suggests that even in patients with spondylolisthesis, the difficulty of spinal anesthesia may not increase significantly if the needle is inserted at the optimal angle.

This study has several limitations. First, the anesthesiologist who performed the ultrasonography was not completely blinded to the group allocation. This was inevitable because structural abnormalities could be visually identified during ultrasound scanning on the patient's back. Second, the accuracy of measurements of small angles or distances with ultrasound is likely inspector-dependent and has some built in error. The possibility of systematic error of measurements exists since the measurements are small. Third, the difference in angulation that we found was only about 3 degrees. Although this is small, the fact that the needle direction is changed from cephalad to caudad is clinically meaningful. The reason for the small difference in angle may be because all of the patients enrolled in this study were low grade spondylolisthesis patients. For patients with spondylolisthesis of a higher grade, the optimal angle of needle insertion may differ, and a larger caudad angulation may be required in them. Fourth, without information about spondylolisthesis from pre-operative lumbosacral X-ray image, it may be difficult to apply the results of this study to the patient. Ultrasonography may be helpful in this situation. Finally, we estimated the optimal angle of needle insertion using an ultrasound probe but did not actually perform spinal puncture. Since this study is observational, further study is necessary to determine the difference in clinical outcomes when this technique is applied on patients.

## Conclusions

In conclusion, anesthesiologists should consider the fact that the optimal angle for needle insertion for spinal anesthesia is more caudad at the level of a spondylolisthesis than at other levels or in patients without spondylolisthesis. This phenomenon is most prominent at the L5/S1 level.

## Data Availability

The datasets used and/or analyzed during the current study are available from the corresponding author on reasonable request.

## Abbreviations

LFD: Ligamentum flavum-dura mater complex
AC: Anterior complex
CT: Computed tomography
MRI: Magnetic resonance imaging
